# Safety of switching to Migalastat from enzyme replacement therapy in Fabry disease: Experience from the Phase 3 ATTRACT study

**DOI:** 10.1002/ajmg.a.61105

**Published:** 2019-03-28

**Authors:** Derralynn A. Hughes, Kathleen Nicholls, Gere Sunder‐Plassmann, Ana Jovanovic, Ulla Feldt‐Rasmussen, Raphael Schiffmann, Robert Giugliani, Vipul Jain, Chris Viereck, Jeffrey P. Castelli, Nina Skuban, Jay A. Barth, Daniel G. Bichet

**Affiliations:** ^1^ Lysosomal Storage Disorders Unit Royal Free NHS Foundation Trust and University College London London United Kingdom; ^2^ Department of Nephrology, Royal Melbourne Hospital University of Melbourne Parkville Victoria Australia; ^3^ Division of Nephrology and Dialysis, Department of Medicine III Medical University of Vienna Vienna Austria; ^4^ The Mark Holland Metabolic Unit Salford Royal Hospital and NHS Foundation Trust Salford United Kingdom; ^5^ Department of Medical Endocrinology and Metabolism Rigshospitalet, University of Copenhagen Copenhagen Denmark; ^6^ Metabolic Disease Baylor Research Institute Dallas Texas; ^7^ Medical Genetics Service, HCPA, and Department of Genetics UFRGS Porto Alegre Rio Grande do Sul Brazil; ^8^ Amicus Therapeutics, Inc. Cranbury New Jersey; ^9^ Departments of Medicine and Physiology, Hôpital du Sacré‐Coeur University of Montreal Montreal Quebec Canada

To the editor:

1

Fabry disease is a rare X‐linked lysosomal storage disorder caused by mutations in the *GLA* gene that result in functional deficiency of alpha‐galactosidase A (α‐Gal A); the accumulation of lysosomal α‐Gal A substrates can lead to multisystem disease and early death (Germain, 2010; Mehta et al., 2010; Waldek, Patel, Banikazemi, Lemay, & Lee, 2009). Until recently, treatment options were limited to enzyme replacement therapy (ERT) with agalsidase alfa or agalsidase beta administered via infusion every 2 weeks (Gaggl & Sunder‐Plassmann, 2016).

Migalastat is a first‐in‐class, small‐molecule pharmacological chaperone that binds to and stabilizes *amenable* mutant forms of α‐Gal A in the endoplasmic reticulum, facilitating proper trafficking to lysosomes, where dissociation of migalastat allows α‐galactosidase to catabolize accumulated substrates (Benjamin et al., 2009; Germain et al., 2016; Germain & Fan, 2009; Ishii et al., 2007; Khanna et al., 2010; Yam, Zuber, & Roth, 2005). It is estimated that 35–50% of patients with Fabry disease have migalastat‐amenable mutations (Hughes et al., 2017). As of July 23, 2018, the total exposure to migalastat in the Phase 2 and 3 clinical programs was 660 patient‐years, with 128 patients exposed ≥1 year (Data on file. Amicus Therapeutics Inc., 2018). The efficacy and safety of migalastat in patients with Fabry disease who have amenable *GLA* mutations have been established in both placebo and active‐controlled clinical trials and long‐term open‐label extension studies (Germain et al., 2016; Germain et al., 2018; Hughes et al., 2017; Nicholls et al., 2018). Oral migalastat has been approved in the European Union, Switzerland, Australia, Israel, Republic of Korea, and Japan for long‐term treatment of adults and adolescents aged 16 years and older with a confirmed diagnosis of Fabry disease (α‐Gal A deficiency) who have a migalastat‐amenable *GLA* mutation (Amicus Therapeutics Inc., 2018). Migalastat is also approved in the United States and Canada for adults (aged 18 years and older; Amicus Therapeutics U.S., Inc., 2018; Amicus Therapeutics UK Ltd., 2017).

We previously reported on Part 1 of the Phase 3 ATTRACT study (AT1001‐012; NCT01218659), an 18‐month randomized treatment comparison that demonstrated comparable efficacy of migalastat (Cohort 1) and ERT (Cohort 2) in male and female patients with Fabry disease previously treated with ERT for >12 months (Hughes et al., 2017). During Part 2 of the ATTRACT study, patients in both cohorts could receive migalastat for an additional 12 months during the optional open‐label extension (OLE). Therefore, both cohorts switched from ERT to migalastat (at baseline for patients randomized at study entry to migalastat [Cohort 1] or month 18 for those randomized to ERT [Cohort 2]). In this article, we assess the safety of switching from ERT to migalastat by evaluating the incidence of adverse events, laboratory assessments, and concomitant medications following switching in both cohorts within the safety population.

At study entry, patients ranged in age from 18 to 72 years with a mean age of 49 years; 56% were female (Hughes et al., 2017). Demographics were balanced between cohorts. Mean time since Fabry diagnosis was 11.4 years, and most patients (88%) had multi‐organ disease (including nervous system [81%], cardiac [71%], gastrointestinal [61%], and renal/urinary [75%] involvement (Hughes et al., 2017); renal/urinary involvement was defined as having any of the following: a medical history of renal or urinary disorders, decreased estimated glomerular filtration rate (eGFR <90 mL/min/1.73 m^2^; ~44% of adults aged 40 to 59 have an eGFR below this cutoff [National Kidney Foundation, 2002]), or 24‐hr urine protein ≥150 mg). Eight patients had a history of premedication for ERT infusion‐associated reactions (IARs; *n* = 5 in Cohort 1; *n* = 3 in Cohort 2); 2 patients in Cohort 2 continued IAR prophylaxis during on‐study ERT. Fifty‐one patients switched from ERT to migalastat: 36 patients in Cohort 1 (Part 1) and 15 patients in Cohort 2 (Part 2/OLE). Most patients continued migalastat treatment until Month 30 (30/36 in Cohort 1 and 12/15 in Cohort 2).

Cohort 1 patients switched to migalastat treatment at baseline, by which time most patients had received >2 years of ERT (mean, 3.5 years; Table 1). Prior ERT characteristics were similar between male and female patients. In patients for whom the data were available, migalastat was started 4 to 19 days after their last ERT infusion. The most common treatment‐emergent adverse events (AEs) in Cohort 1 (occurring in ≥20% of patients) during the first 18 months were nasopharyngitis (33%) and headache (25%; Table 2), and during the full 30 months were nasopharyngitis (42%), headache (36%), and influenza (27%; Table 2). AEs were generally mild or moderate; no patient discontinued due to an AE. There were no clinically meaningful changes in mean values from baseline for hematology, serum chemistry, urinalysis analysis, and vital signs (Supporting Information Table S1). Thirty‐four (94%) Cohort 1 patients started a new medication during months 0–30. The most common new concomitant mediations were amoxicillin (22%), ibuprofen (19%), paracetamol (19%), amoxicillin with clavulanic acid (11%), and temazepam (11%). Only two (6%) patients started a new angiotensin‐converting enzyme inhibitor, angiotensin II receptor blocker, or renin inhibitor. Overall, based on a review of AEs, laboratory measures, and concomitant medications in Cohort 1, migalastat was well tolerated after patients switched from ERT.

**Table 1:** Enzyme replacement therapy characteristics prior to start of Migalastat

	Cohort 1^a^			Cohort 2^b^		
	Female	Male	Overall	Female	Male	Overall
ERT at baseline, *n* (%)						
Agalsidase beta	6 (30.0)	5 (31.3)	11 (30.6)	5 (50.0)	0	5 (33.3)
Agalsidase alfa	14 (70.0)	10 (62.5)	24 (66.7)	5 (50.0)	5 (100.0)	10 (66.7)
Missing	0	1 (6.3)	1 (2.8)	0	0	0
ERT duration, years						
*N*	17	12	29	10	5	15
Mean ± *SD*	3.0 ± 2.1	4.2 ± 3.7	3.5 ± 2.9	4.9 ± 3.0	6.0 ± 3.1	5.2 ± 3.0
Median	2.2	2.8	2.3	3.6	6.9	3.6
(min, max)	(1.0, 8.4)	(0.4, 12)	(0.4, 12)	(1.3, 10)	(2.3, 9.9)	(1.3, 10)
ERT duration, *n* (%)						
<2 years	6 (30.0)	3 (18.8)	9 (25.0)	2 (20.0)	0	2 (13.3)
2 to 3 years	7 (35.0)	3 (18.8)	10 (27.8)	0	1 (20.0)	1 (6.7)
3 to 4 years	1 (5.0)	2 (12.5)	3 (8.3)	4 (40.0)	1 (20.0)	5 (33.3)
>4 years	3 (15.0)	4 (25.0)	7 (19.4)	4 (40.0)	3 (60.0)	7 (46.7)
Missing	3 (15.0)	4 (25.0)	7 (19.4)	0	0	0

*Note*. ERT = enzyme replacement therapy; max = maximum; min = minimum; n = number of patients with data; SD = standard deviation.

a Thirty‐six patients who were randomized to receive migalastat treatment.

b For Cohort 2, only patients in the open‐label extension phase were included.

**Table 2:** Treatment‐emergent adverse events occurring in ≥10% of Cohort 1 (Migalastat‐Migalastat) patients

AE (preferred term), *n* (%)	Part 1: Migalastat (0–18 months)^d^ (*n* = 36)^a^	Parts 1 and 2: Migalastat (0–30 months)^b^ (*n* = 33)^c^
Nasopharyngitis	12 (33)	14 (42)
Headache	9 (25)	12 (36)
Influenza	5 (14)	9 (27)
Cough	3 (8)	6 (18)
Diarrhea	5 (14)	6 (18)
Nausea	5 (14)	6 (18)
Dizziness	6 (17)	5 (15)
Abdominal pain	5 (14)	5 (15)
Urinary tract infection	4 (11)	5 (15)
Blood creatine phosphokinase increased	3 (8)	5 (15)
Myalgia	3 (8)	5 (15)
Arthralgia	3 (8)	4 (12)
Pyrexia	3 (8)	4 (12)
Sinusitis	3 (8)	4 (12)
Vomiting	3 (8)	4 (12)
Protein urine present	3 (8)	4 (12)
Pain	1 (3)	4 (12)
Back pain	4 (11)	3 (9)
Upper respiratory tract infection	4 (11)	3 (9)

*Note*. AE = adverse event; OLE = open‐label extension.

a Number of patients who had at least one dose of migalastat during Part 1. Thirty‐four patients completed treatment through 18 months (Part 1). The reason for discontinuation during Part 1 was withdrawal by participant (*n* = 2).

b Any adverse events that started on or after randomized treatment period first dose date up to 30 days after OLE last dose date.

c Number of patients who had at least one dose of migalastat during Part 2. Thirty patients completed treatment through 30 months (Part 2). Reasons for discontinuation during Part 2 include withdrawal by participant (*n* = 1), pregnancy (*n* = 1), and lack of efficacy (*n* = 1).

Any adverse events that started after first study drug administration and before OLE first dose date.

Cohort 2 patients switched treatment at Month 18, after most patients completed >3 years of ERT (mean, 5.2 years; Table 1). Nasopharyngitis (33%), headache (24%), and cough (24%) were the most common AEs during the 18‐month ERT treatment period in Part 1 (Table 3). In patients for whom the data were available, migalastat was started 2 to 14 days after their last ERT infusion. The most common AEs during 12 months of migalastat treatment in Part 2 were nasopharyngitis (33%), diarrhea (27%), vomiting (27%), influenza (20%), and headache (20%; Table 3). Although the percentages of patients experiencing diarrhea or vomiting increased after the switch to migalastat, these reflect changes in only 1–2 patients and the small patient numbers limit interpretation. There were no clinically meaningful changes in mean values from hematology, serum chemistry, urinalysis analysis, and vital signs following the switch from ERT to migalastat (Supporting Information Table S2). Twelve (80%) Cohort 2 patients started a new medication during months 18–30. The most common new concomitant medications were general anesthetics (13%), clindamycin (13%), ibuprofen (13%), naproxen (13%), and paracetamol (13%). Only 1 (7%) patient started a new angiotensin‐converting enzyme inhibitor, angiotensin II receptor blocker, or renin inhibitor. Review of AEs, laboratory measures, and concomitant medications in Cohort 2 did not identify notable safety concerns after switching to migalastat.

**Table 3:** Treatment‐emergent adverse events occurring in ≥10% of cohort 2 (ERT‐Migalastat) patients

AE (preferred term), *n* (%)	Part 1: ERT (0–18 months)^a^ (*n* = 21)^b^	Part 2: Migalastat (18–30 months)^c^ (*n* = 15)^d^
Nasopharyngitis	7 (33)	5 (33)
Diarrhea	2 (10)	4 (27)
Vomiting	3 (14)	4 (27)
Headache	5 (24)	3 (20)
Influenza	4 (19)	3 (20)
Abdominal pain	2 (10)	2 (13)
Arthralgia	2 (10)	2 (13)
Bronchitis	3 (14)	2 (13)
Dizziness	2 (10)	2 (13)
Nausea	2 (10)	2 (13)
Blood creatine phosphokinase increased	1 (5)	2 (13)
Fatigue	1 (5)	2 (13)
Neuralgia	1 (5)	2 (13)
Pyrexia	1 (5)	2 (13)
Diabetes mellitus	0	2 (13)
Muscle spasm	0	2 (13)
Poor quality sleep	0	2 (13)
Cough	5 (24)	1 (7)
Back pain	3 (14)	1 (7)
Dyspnea	2 (10)	1 (7)
Pain in extremity	2 (10)	1 (7)
Sinusitis	3 (14)	0
Dry mouth	2 (10)	0
Gastritis	2 (10)	0
Peripheral edema	2 (10)	0
Procedural pain	2 (10)	0
Vertigo	2 (10)	0

*Note*. AE = adverse event; ERT = enzyme replacement therapy; OLE = open‐label extension.

a Any adverse events that started after first study drug administration and before OLE first dose date.

b Number of patients who had at least one dose of ERT during the study. Eighteen patients completed treatment through 18 months (Part 1); the reason for discontinuation during Part 1 was withdrawal by participant (*n* = 3).

c Any adverse events that started on or after randomized treatment period first dose date up to 30 days after OLE last dose date.

d Number of patients who had at least one dose of migalastat during Part 2. Twelve patients completed treatment through 30 months (Part 2). Reasons for discontinuation during Part 2 include withdrawal by participant (*n* = 1), physician decision (*n* = 1), lost to follow‐up (*n* = 1).

These data demonstrated a favorable safety profile after patients directly switched to migalastat 150 mg QOD 2 to 19 days following their last ERT infusion. Migalastat was generally well tolerated, and no patients discontinued treatment due to AEs. Reason for discontinuing ERT during 0–18 months was withdrawal by participant (*n* = 3); reasons for discontinuing migalastat during 0–30 months were withdrawal by participant (*n* = 4), pregnancy (*n* = 1), lack of efficacy (*n* = 1), physician decision unrelated to migalastat (*n* = 1), and lost to follow‐up (*n* = 1).

Limitations of the analysis include the relatively small number of patients who switched from agalsidase beta to migalastat, as most (67%) patients switched from agalsidase alfa because enrollment for ATTRACT coincided with the worldwide shortage of agalsidase beta.

Migalastat is the only oral treatment for Fabry disease, which provides a suitable alternative to once‐every‐2‐weeks intravenous ERT in patients with amenable mutations who are ERT‐experienced and can also be utilized as a first‐line therapy in ERT‐naive patients. Although there has not yet been a consensus among physicians who treat patients with Fabry disease on when to choose migalastat over ERT, we have developed some criteria in our clinical practices, which include: age 16 years and older (18 years and older in the United States and Canada), a confirmed amenable mutation, an eGFR > 30 mL/min/1.73 m^2^, compliance with every‐other‐day oral administration, and no intention by female patients to become pregnant. Patients' preference and hypersensitivity to ERT are also factors in considering the best treatment option for patients. We suggest having a comprehensive counseling session with the patient to discuss the mechanism of action, clinical data, and approved indication for migalastat, as well as schedule of administration. For patients switching from ERT, migalastat is commonly initiated ~2 weeks after the last dose of ERT based on the infusion interval; however, other practical considerations may influence the exact duration between the last ERT infusion and first dose of migalastat. Migalastat may be safely initiated within days of the last ERT infusion.

In conclusion, patients with amenable mutations who have been receiving ERT infusions can be safely switched to migalastat 150 mg QOD, and no special procedure is needed for the switch.

## AUTHOR CONTRIBUTIONS

The study was designed by the sponsor (Amicus) and a core group of investigators. Data collection and analyses were undertaken by the sponsor (Amicus) in collaboration with investigators. The first draft of the manuscript was written by the first author with medical writing assistance provided by Brian Zeiler. All authors critically reviewed drafts of the manuscript. All authors vouch for the completeness and accuracy of the data and analyses and for the fidelity of the study to the protocol. All authors made the decision to submit the manuscript for publication.

## CONFLICT OF INTEREST

DAH has served as a consultant for and received research funding and honoraria from Amicus, Shire, Sanofi Genzyme, Protalix, and Actelion. KN has served as an advisor for Amicus, Shire, and Sanofi Genzyme, has received research support from Amicus and Shire, and has received travel support from Sanofi Genzyme. GS‐P has received personal fees and non‐financial support from Amicus and grant funding, personal fees, and non‐financial support from Shire and Sanofi Genzyme. AJ has received advisory honoraria and speaker's fees from Amicus, Shire, Biomarin and Sanofi Genzyme. UFR reports other support from Amicus during the conduct of the study, grant support and speaker's honoraria from Amicus, Sanofi Genzyme, and Shire outside the submitted work, and research funding from Novo Nordisk Research Foundation. RS has served as a consultant for and received research funding from Amicus and Protalix Biotherapeutics. RG has received honoraria from Amicus, Biomarin, Sanofi Genzyme, and Shire. CV, JPC, NS, and JAB are employees of and hold stock in Amicus. DGB has received research funding, serves as a consultant, and is on the speaker's bureau for Amicus and Sanofi Genzyme, and has received research funding from Shire.

## Supporting information


**Table S1** Change in Laboratory Measurements and Vital Signs After Switch From ERT to Migalastat in Cohort 1
**Table S2.** Change in Laboratory Measurements and Vital Signs After Switch From ERT to Migalastat in Cohort 2
