# Supplementary material for: Safety of switching to Migalastat from enzyme replacement therapy in Fabry disease: Experience from the Phase 3 ATTRACT study
Source: Am J Med Genet A. 2019 Mar 28;179(6):1069–73. doi: 10.1002/ajmg.a.61105 (PMC6593787; doi:10.1002/ajmg.a.61105)
Supplement: Supplementary file 1 — Table S1 Change in Laboratory Measurements and Vital Signs After Switch From ERT to Migalastat in Cohort 1 Table S2. Change in Laboratory Measurements and Vital Signs After Switch From ERT to Migalastat in Cohort 2 [file AJMG-179-1069-s001.docx]

**Table S1. Change in Laboratory Measurements and Vital Signs After Switch From ERT to Migalastat in Cohort 1**

|  | **n** | **Change from baseline to Month 18**  **mean ± SD** | **Normal at baseline**  **n (%)** | **Normal at Month 18**  **n (%)** |
| --- | --- | --- | --- | --- |
| **Hematology** | | | | |
| Basophils, 10^9^/L | 34 | 0.0 ± 0.0 | 34 (100) | 34 (100) |
| Basophils/leukocytes, % | 34 | 0.1 ± 0.3 | 34 (100) | 34 (100) |
| Eosinophils, 10^9^/L | 34 | 0.0 ± 0.1 | 34 (100) | 34 (100) |
| Eosinophils/leukocytes, % | 34 | 0.1 ± 1.1 | 32 (94) | 33 (97) |
| Erythrocytes, 10^12^/L | 34 | 0.1 ± 0.3 | 30 (88) | 29 (85) |
| Hematocrit, % | 34 | 0.3 ± 2.6 | 27 (79) | 29 (85) |
| Hemoglobin, g/dL | 34 | -0.1 ± 0.8 | 29 (85) | 29 (85) |
| Leukocytes, 10^9^/L | 34 | 0.5 ± 1.9 | 32 (94) | 32 (94) |
| Lymphocytes, 10^9^/L | 34 | 0.1 ± 0.3 | 33 (97) | 33 (97) |
| Lymphocytes/leukocytes, % | 34 | 0.0 ± 6.0 | 33 (97) | 34 (100) |
| Monocytes, 10^9^/L | 34 | 0.0 ± 0.1 | 33 (97) | 34 (100) |
| Monocytes/leukocytes, % | 34 | 0.5 ± 1.2 | 33 (97) | 33 (97) |
| Neutrophils, 10^9^/L | 34 | 0.1 ± 1.2 | 32 (94) | 31 (91) |
| Neutrophils/leukocytes, % | 34 | -0.8 ± 7.1 | 32 (94) | 32 (94) |
| Platelets, 10^9^/L | 34 | 13.0 ± 38.1 | 33 (97) | 34 (100) |
| **Chemistry** | | | | |
| Alanine aminotransferase, IU/L | 34 | 1.4 ± 10.3 | 32 (94) | 32 (94) |
| Albumin, g/dL | 36 | 0.1 ± 0.2 | 36 (100) | 35 (97) |
| Alkaline phosphatase, IU/L | 36 | -1.4 ± 16.8 | 32 (89) | 31 (86) |
| Aspartate aminotransferase, IU/L | 34 | 1.1 ± 6.4 | 33 (97) | 32 (94) |
| Bicarbonate, mmol/L | 36 | 0.4 ± 2.9 | 31 (86) | 32 (89) |
| Bilirubin, mg/dL | 36 | 0.0 ± 0.1 | 34 (94) | 34 (94) |
| Blood urea nitrogen, mmol/L | 36 | 0.3 ± 1.3 | 36 (100) | 35 (97) |
| Calcium, mmol/L | 36 | 0.0 ± 0.1 | 35 (97) | 36 (100) |
| Chloride, mmol/L | 36 | -1.2 ± 3.3 | 36 (100) | 36 (100) |
| Creatine kinase, IU/L | 35 | 23.5 ± 63.5 | 33 (94) | 28 (80) |
| Creatinine, mg/dL | 36 | 0.0 ± 0.1 | 34 (94) | 33 (92) |
| Gamma-glutamyl transferase, IU/L | 35 | 0.3 ± 21.0 | 32 (91) | 33 (94) |
| Glucose, mmol/L | 36 | -0.2 ± 1.7 | 33 (92) | 33 (92) |
| Lactate dehydrogenase, IU/L | 31 | 13.3 ± 92.0 | 27 (87) | 23 (74) |
| Magnesium, mmol/L | 35 | 0.0 ± 0.0 | 34 (97) | 33 (94) |
| Phosphate, mmol/L | 35 | 0.1 ± 0.2 | 34 (97) | 34 (97) |
| Potassium, mmol/L | 35 | 0.1 ± 0.5 | 35 (97) | 33 (94) |
| Protein, g/dL | 36 | 0.0 ± 0.4 | 36 (100) | 35 (97) |
| Sodium, mmol/L | 36 | -0.7 ± 2.5 | 35 (97) | 36 (100) |
| Uric acid, µmol/L | 36 | 2.4 ± 39.1 | 34 (94) | 36 (100) |
| **Urinalysis** | | | | |
| Specific gravity | 36 | 0.0 ± 0.0 | 36 (100) | 35 (97) |
| pH | 36 | 0.1 ± 0.7 | 36 (100) | 36 (100) |
| **Vital signs** | | | | |
| Systolic blood pressure, mm Hg | 36 | -3.2 ± 15.5 | NA | NA |
| Diastolic blood pressure, mm Hg | 36 | -2.0 ± 11.8 | NA | NA |
| Weight, kg | 36 | -0.9 ± 6.9 | NA | NA |
| Pulse rate, bpm | 35 | 1.3 ± 10.5 | NA | NA |
| Respiration rate, breaths/min | 34 | -0.3 ± 2.2 | NA | NA |
| Body mass index, kg/cm^2^ | 36 | -0.4 ± 2.5 | NA | NA |

bpm = beats per minute; NA = not analyzed.

**Table S2. Change in Laboratory Measurements and Vital Signs After Switch From ERT to Migalastat in Cohort 2**

|  | **n** | **Change from Month 18 to Month 30**  **mean ± SD** | **Normal at baseline**  **n (%)** | **Normal at Month 30**  **n (%)** |
| --- | --- | --- | --- | --- |
| **Hematology** | | | | |
| Basophils, 10^9^/L | 14 | 0.0 ± 0.0 | 14 (100) | 14 (100) |
| Basophils/leukocytes, % | 14 | 0.0 ± 0.3 | 14 (100) | 14 (100) |
| Eosinophils, 10^9^/L | 14 | 0.0 ± 0.1 | 14 (100) | 14 (100) |
| Eosinophils/leukocytes, % | 14 | -0.1 ± 1.1 | 14 (100) | 13 (93) |
| Erythrocytes, 10^12^/L | 14 | 0.1 ± 0.4 | 13 (93) | 13 (93) |
| Hematocrit, % | 14 | 1.9 ± 3.4 | 11 (79) | 13 (93) |
| Hemoglobin, g/dL | 14 | 0.4 ± 1.0 | 12 (86) | 12 (86) |
| Leukocytes, 10^9^/L | 14 | -0.2 ± 1.0 | 14 (100) | 13 (93) |
| Lymphocytes, 10^9^/L | 14 | 0.0 ± 0.6 | 14 (100) | 11 (79) |
| Lymphocytes/leukocytes, % | 14 | 0.7 ± 10.4 | 14 (100) | 13 (93) |
| Monocytes, 10^9^/L | 14 | 0.0 ± 0.1 | 13 (93) | 12 (86) |
| Monocytes/leukocytes, % | 14 | 0.2 ± 2.9 | 13 (93) | 13 (93) |
| Neutrophils, 10^9^/L | 14 | -0.2 ± 1.3 | 14 (100) | 13 (93) |
| Neutrophils/leukocytes, % | 14 | -0.7 ± 11.2 | 14 (100) | 14 (100) |
| Platelets, 10^9^/L | 13 | -14.5 ± 28.2 | 13 (100) | 12 (92) |
| **Chemistry** | | | | |
| Alanine aminotransferase, IU/L | 14 | 3.1 ± 9.9 | 14 (100) | 11 (79) |
| Albumin, g/dL | 14 | 0.0 ± 0.3 | 13 (93) | 14 (100) |
| Alkaline phosphatase, IU/L | 14 | 5.3 ± 12.4 | 13 (93) | 12 (86) |
| Aspartate aminotransferase, IU/L | 14 | 0.1 ± 9.4 | 14 (100) | 13 (93) |
| Bicarbonate, mmol/L | 14 | 0.8 ± 3.3 | 13 (93) | 10 (71) |
| Bilirubin, mg/dL | 14 | 0.1 ± 0.2 | 10 (71) | 12 (86) |
| Blood urea nitrogen, mmol/L | 14 | 0.8 ± 1.6 | 13 (93) | 11 (71) |
| Calcium, mmol/L | 14 | 0.0 ± 0.1 | 14 (100) | 12 (86) |
| Chloride, mmol/L | 14 | -1.5 ± 3.0 | 14 (100) | 13 (93) |
| Creatine kinase, IU/L | 14 | -47.4 ± 119.5 | 14 (100) | 13 (93) |
| Creatinine, mg/dL | 14 | 0.0 ± 0.2 | 13 (93) | 12 (86) |
| Gamma-glutamyl transferase, IU/L | 14 | 0.1 ± 9.3 | 10 (71) | 9 (64) |
| Glucose, mmol/L | 14 | 1.1 ± 4.3 | 13 (93) | 12 (86) |
| Lactate dehydrogenase, IU/L | 14 | 0.9 ± 27.7 | 12 (86) | 10 (71) |
| Magnesium, mmol/L | 14 | 0.0 ± 0.0 | 13 (93) | 14 (100) |
| Phosphate, mmol/L | 14 | -0.1 ± 0.2 | 14 (100) | 14 (100) |
| Potassium, mmol/L | 14 | 0.0 ± 0.4 | 13 (93) | 14 (100) |
| Protein, g/dL | 14 | 0.0 ± 0.5 | 13 (93) | 13 (93) |
| Sodium, mmol/L | 14 | -0.7 ± 2.7 | 14 (100) | 12 (86) |
| Uric acid, µmol/L | 14 | 16.1 ± 79.0 | 13 (93) | 12 (86) |
| **Urinalysis** | | | | |
| Specific gravity | 14 | 0.0 ± 0.0 | 14 (100) | 13 (93) |
| pH | 14 | -0.2 ± 0.4 | 14 (100) | 14 (100) |
| **Vital signs** | | | | |
| Systolic blood pressure, mm Hg | 14 | 4.4 ± 10.6 | NA | NA |
| Diastolic blood pressure, mm Hg | 14 | 1.9 ± 8.7 | NA | NA |
| Weight, kg | 14 | 0.5 ± 5.3 | NA | NA |
| Pulse rate, bpm | 14 | 5.0 ± 10.7 | NA | NA |
| Respiration rate, breaths/min | 14 | 0.0 ± 3.4 | NA | NA |
| Body mass index, kg/cm^2^ | 14 | 0.1 ± 2.0 | NA | NA |

bpm = beats per minute; NA = not analyzed.
